# Work-related stress perception and hypertension amongst health workers of a mission hospital in Oyo State, south-western Nigeria

**DOI:** 10.4102/phcfm.v4i1.307

**Published:** 2012-04-19

**Authors:** Akinwumi O. Owolabi, Mojisola O. Owolabi, Akintayo D. OlaOlorun, Ayo Olofin

**Affiliations:** 1 Family Medicine Department, Federal Medical Centre Asaba, Delta State, Nigeria; 2 Family Medicine Department, Baptist Medical Centre Ogbomoso, Oyo State, Nigeria; 3 Staff Medical Services Department, University College Hospital, Ibadan, Oyo State, Nigeria

## Abstract

**Background:**

Globalisation and changes in the nature of work have resulted in increasing work-related stress in people in developing countries. Work stress is at present already acknowledged as one of the epidemics of modern working life. It is associated with a number of disease conditions, such as hypertension, cardiovascular diseases, affective disorders, depression, disturbed metabolism (risk of Type II diabetes) and musculoskeletal disorders.

**Objective:**

This study was a work site cross-sectional descriptive study carried out amongst the health workers at the Baptist Medical Centre Ogbomoso, Oyo State, south-western Nigeria. The aim of the study was to discern the prevalence of perceived work stress and to explore the relationship between perceived work stress and the presence of hypertension.

**Methods:**

A total of 324 consenting health workers of the institution were administered the job demand-control questionnaire to assess work stress. A standardised questionnaire was used to collect socio-demographic data and other personal data. Measurements of blood pressure, weight and height were carried out and body mass indices were calculated.

**Results:**

More than a quarter (26.2%) of the subjects perceived themself as stressed at work. The single largest group of hypertensive subjects was seen amongst subjects with work stress.

**Conclusion:**

A significant number of health workers in this study is afflicted by work-related stress and perceived work stress was found to be significantly associated with higher hypertension prevalence.

## Abstrait

**La perception du stress lié au travail et l'hypertension chez le personnel soignant d'un hôpital missionnaire dans l'Etat d'Oyo, au Sud-ouest du Nigeria**

**Présentation:**

La mondialisation et les changements dans la nature du travail ont donné lieu à un stress lié au travail chez les habitants des pays en voie de développement. A l'heure actuelle, le stress lié au travail est déjà reconnu comme l'une des épidémies de la vie professionnelle moderne. Il est associé à plusieurs troubles de la santé tels que l'hypertension, les maladies cardiovasculaires, les troubles de l'humeur, la dépression, les troubles du métabolisme (risque de diabète de type II) et les troubles musculo-squelettiques.

**Objectif:**

Cette étude a consisté en une étude descriptive transversale d'un lieu de travail a été réalisée auprès des professionnels de la santé au Centre médical baptiste d'Ogbomoso, dans l'Etat d'Oyo, au Sud-ouest du Nigeria. L'objectif de l’étude était de déterminer la prévalence du stress lié au travail perçu et d’étudier la relation entre le stress lié au travail perçu et l'hypertension.

**Méthodes:**

Au total, 324 travailleurs de la santé consentants de l'institution ont répondu au questionnaire sur les exigences et le contrôle associés au poste en vue de l’évaluation du stress lié au travail. Un questionnaire standardisé a été utilisé afin de recueillir les données sociodémographiques et autres données personnelles. Des mesures de la tension artérielle, du poids et de la taille ont été prises et les indices de masse corporelle ont été calculés.

**Résultats:**

Plus d'un quart (26.2%) des sujets se percevaient comme stressés au travail. Le grand groupe de sujets hypertensifs le plus important a été observé chez ceux souffrant de stress lié au travail.

**Conclusion:**

Un nombre significatif de travailleurs de la santé ayant participé à cette étude sont touchés par le stress lié au travail et le stress lié au travail perçu était significativement associé à une plus forte prévalence de l'hypertension.

## Introduction

### Background

Traditionally, the focus of occupational health and safety initiatives has been on chemical, biological and physical exposures, whilst the psychosocial risks at work are still largely neglected and their causes and consequences still insufficiently understood as they pertain to the developing country context.^1^ Unlike physical or chemical hazards, work stress has no obvious tangible hazardous agent.

Work-related stress follows a pattern of reactions that occurs when workers are presented with work demands not matched to their knowledge, skills or abilities and which challenge their ability to cope.^1^ It is defined as high psychological demands and low decision latitude on the job.^2, 3^ Job strain, defined as a measure of the balance between the psychological demands of a job and the amount of control or decision-making power it affords, has been proposed as a key component of work stress.^3–5^

The ‘job strain’ model proposed by Karasek states that the combination of high job demands and low job decision latitude (high job strain or work stress) will lead to negative physical health outcomes such as hypertension and cardiovascular disease.^5, 6^ According to a World Health Organisation publication,^1^ hypertension and other cardiovascular diseases are amongst the main chronic diseases in the developed and developing countries and consume an important proportion of their public health budget. It is estimated to affect about 20% of the adult population in most countries of the world, and accounts for 20% – 50% of all deaths.^1^

The proposed mechanisms through which job strain contributes to high blood pressure and consequently to cardiovascular disease, is through chronic physiological arousal with the stimulation of the sympathetic nervous system.^7^ This causes an increase in peripheral resistance which results in raised blood pressure. Stress may also operate on a background of genetic susceptibility and interact with other lifestyle determinants of hypertension such as obesity, physical inactivity, over-eating, smoking, excessive salt intake and excessive alcohol consumption.^7^ The extent, however, to which psychological stress contributes to raise blood pressure is still uncertain.^7^

### Objective

Work stress has been implicated as an independent risk factor in the aetiology of coronary heart disease and increased hypertensive risk in a number of occupations.^8, 9^ High job strain could be contributing from 21% to 32% of hypertension prevalence.^1^ There is a paucity of literature on the health sector's workplace stress and the prevalence of hypertension in Africa. The aim of this study, therefore, was to determine the prevalence of perceived work stress and to explore the relationship between perceived work stress and the presence of hypertension amongst the health workers of the Baptist Medical Centre, Ogbomoso.

Ogbomoso is a town located about 100 km north of Ibadan, the capital of Oyo State, in the south-western part of Nigeria. The Baptist Medical Centre is a 200-bed mission hospital, run by 351 health workers. It was founded in 1907 and it provides primary, secondary and tertiary health-care services.

Occupational stress has been a long-standing concern of the health-care industry and studies from developed countries indicate that health-care workers have higher rates of substance abuse, suicide and elevated rates of depression and anxiety, linked to job stress, than other professionals.^9^ This makes the health-care industry suitable for studies that examine the association between the presence of occupational stress and the occurrence of hypertension in people who work in the industry.

## Ethical considerations

Approval was obtained from the hospital's committee on the ethics of human experimentation before the commencement of the study, which was conducted between July 2008 and January 2009. Written consent was obtained from each of the subjects before they were included in the study.

## Methods

### Design and setting

The study was a cross-sectional descriptive study. The study was carried out in the workplace and the subjects were the health workers of the Baptist Medical Centre, Ogbomoso, Oyo State. All consenting health workers of the institution were enrolled for the study. All hypertensive staff with reported history of secondary hypertension and all pregnant women were excluded from the study.

### Samples and population

The age, gender, marital status, religion, nationality, occupation, educational status, physical activity, family type, history of alcohol consumption or cigarette smoking, history of hypertension, family history of hypertension, history of diabetes, ethnic group, and level of education were obtained from the subjects by using a structured questionnaire.

### Procedure

The blood pressure of each subject was measured by using the same mercury sphygmomanometer with a cuff size of 12.5 cm and a stethoscope (Littmann^®^ USA). The left arm of the subjects was used, with the patient in the sitting position after 5 minutes of rest. The cuff of the sphygmomanometer was applied evenly and snugly around the bare arm with the lower edge at least 2.5 cm above the ante-cubital fossa.^10, 11^

The cuff was inflated rapidly to approximately 30 mmHg above the level at which the radial pulse was no longer palpable. Thereafter the cuff was deflated slowly whilst listening with a stethoscope placed over the brachial artery in the ante-cubital fossa. The onset of the first tapping sound (Phase I) was taken as the systolic pressure, whilst the point of complete disappearance of the sound (Phase V) was taken as the diastolic pressure for each subject.^11^ The blood pressure of the subjects was measured on two different occasions at least 5 minutes apart and the average was used for all the subjects.^10, 12, 13^ Hypertension was defined as a systolic blood pressure of ≥ 140 mmHg and/or a diastolic blood pressure of ≥ 90 mmHg.

Body weight was determined with a standardised bathroom weighing scale (Hugo^®^ China) that was calibrated daily. Subjects were weighed barefooted in light clothing, and the same weighing scale was used for all the subjects and readings were expressed to the nearest 0.5 kg. The body mass index (BMI) (kg/m^2^) was used to define obesity.

The BMI was calculated by using the formula^12, 13^

Eqn. 1 $$\text{BMI}=\text{Weight}(\text{kg})/(\text{Height squared})(m^{2})$$

The BMI (kg/m^2^) was classified as follows: BMI < 18.5 (i.e. Underweight), BMI 18.5–24.9 (i.e. Normal weight), BMI 25.0–29.9 (i.e. Overweight), and BMI ≥ 30.0 (i.e. Obese).

Perceived job stress was measured by using the ‘job demand-control questionnaire,‘ based on the job strain model proposed by Karasek in 1979.^2–6^ The questionnaire, which was in English, was also translated into Yoruba and pre-tested to ensure its validity. The self-administered questionnaire contained 11 items that concerned the psychosocial aspects of work, each graded on a 4-point scale. The items were selected from several sources, based on its particular suitability for measuring job stress in a heterogeneous population.^6, 7, 14–16^

Two psychosocial work indices were used: psychological demands (a 5-item indicator measuring job demands, time pressures, and conflicting demands, total score of 5 to 20), and decision latitude (a 6-item indicator measuring influence or control over work, job variety, and the possibilities for learning new skills, a total score of 6 to 24).

Median values of job demand and job control latitude was used to divide the subjects into four groups, namely: the high strain group, the active group, the passive group and the low strain group. Subjects displaying a job demand higher than the median and a job decision latitude lower than the median, represented the high-strain group. Subjects reporting a high decision latitude and a high job demand were in the active group. Subjects with a low job demand and low decision latitude were in the passive group. Subjects with a low job demand and a high decision latitude were in the low-strain group.^2, 3, 7, 15–17^

High-strain, the combination of high job demands and a low job decision latitude is reported to often lead to negative physical health outcomes such as hypertension and cardiovascular disease. It is important to mention that the generalisability of the job demand-control questionnaire makes it possible to draw comparisons amongst different medical and non-medical occupational groups and this was an important factor in selecting the questionaire.^3, 8, 17^

Health workers were grouped according to the World Health Organisation Global Atlas of the Health Workforce (Health Workforce: Aggregated data).^18^ The aggregated data set was used to classify the health workforce according to the following broad categories:

- **Physicians, Dentists:** includes generalists and specialists
- **Nursing and midwifery personnel:** includes professional nurses, professional midwives, auxiliary nurses, auxiliary midwives, enrolled nurses, enrolled midwives and other personnel, such as dental nurses and primary care nurses. Traditional-birth attendants are not counted here, but are included as community and/or traditional-health workers (see below).
- **Pharmaceutical personnel:** includes pharmacists, pharmaceutical assistants, pharmaceutical technicians and related occupations
- **Laboratory health workers:** includes laboratory scientists, laboratory assistants, laboratory technicians, radiographers and related occupations
- **Other health workers:** includes a large range of other cadres of health service providers such as medical assistants, dieticians and nutritionists, occupational therapists, operators of medical and dentistry equipment, optometrists and opticians, physiotherapists, podiatrists, personal care workers, psychologists, respiratory therapists, speech pathologists, and medical trainees and interns
- **Health management and support workers:** includes other categories of health systems personnel, such as managers of health and personal-care services, health economists, health statisticians, health policy lawyers, medical records and health information technicians, ambulance drivers, building-maintenance staff, and other general management and support staff.

### Analysing

The data were analysed with a computer. The Statistical Package for the Social Science, version 11, (SPSS 11) software was used for analysis. The median values for job demand and job control were calculated. The Chi-square test was performed to assess the relationship between work stress and the prevalence of hypertension. Chi-square tests were also performed to analyse the relationship between the other variables. A *p*-value < 0.05 was set as the level of statistical significance.

## Results

Out of the 351 health workers of the institution, 324 health workers consented to, and fulfilled the inclusion criteria for the study. The study group comprised 143 (44.1%) male subjects and 181 (55.9%) female subjects (Table 1). The range of the subjects’ ages was 20–65 with a mean age of 41.1 ± 10.1 years standard deviation. The age range 40–49 years had the highest percentage of subjects in the study (35.8%), whilst there was a lower representation at the extreme age ranges. The majority of the subjects were from the Yoruba ethnic group (94.1%) and most of them were married (83.1%). The study population was mainly Christian (99.4%) whilst the rest was Muslim (0.6%). More than half of the study population had tertiary education (62.6%), and only a few had no formal education (0.3%). The occupational groups were: Health management and support workers, Nurses, Laboratory Personnel, Pharmacy personnel, Physicians and other health workers.

**TABLE 1:** Socio-demographic characteristics of the subjects.

Variables		*n*	%
Age (years)	20–29	49	15.1
	30–39	90	27.8
	40–49	116	35.8
	50–59	55	17.0
	60–65	14	4.3
Sex	Male	143	41.1
	Female	181	59.9
Ethnicity	Yoruba	305	94.1
	Others (Ibo, Ishan, Urhobo, Bini, Fulani)	19	5.9
Marital status	single	50	15.4
	married	269	83.1
	separated	4	1.2
	widowed	1	0.3
Religion	Christianity	322	99.4
	Islam	2	0.6
Nationality	Nigerian	324	100.0
Level of Education	No formal education	1	0.3
	Primary education	29	9.0
	Secondary education	91	28.1
	Tertiary education	203	62.6
Occupational groups	Physicians	18	5.6
	Nurses	81	25.0
	Pharmacy personnel	22	6.8
	Laboratory personnel	28	8.6
	Other health workers	78	24.1
	Health management and support workers	97	29.9

*n*, Number of subjects affected by variable.

**FIGURE 1:**
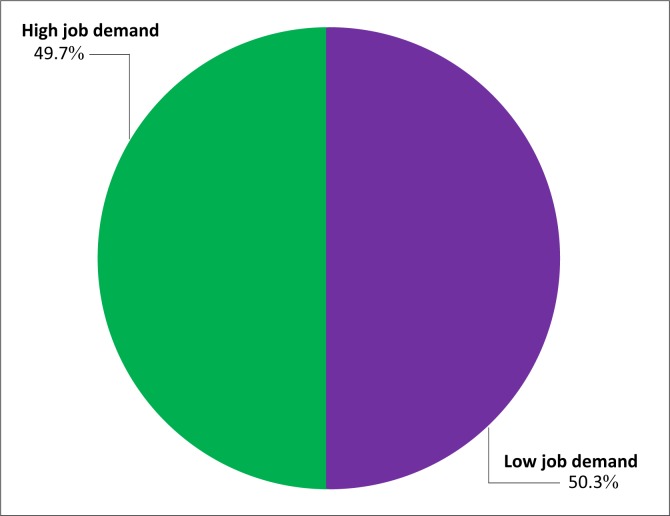
Distribution of subjects by job demand.

**FIGURE 2:**
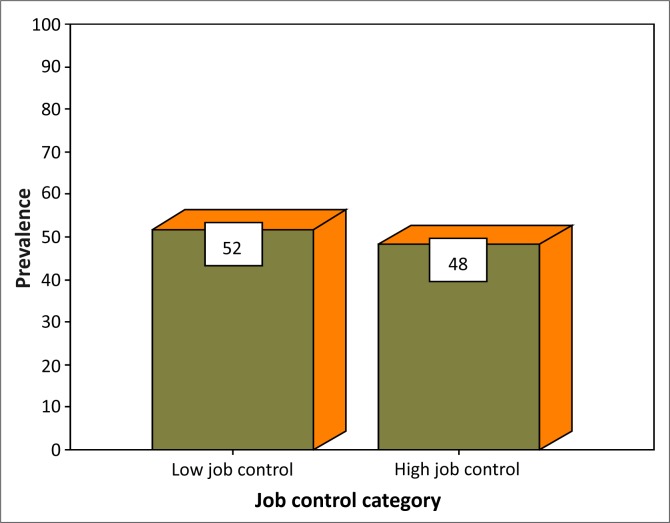
Distribution of subjects by job control.

The distribution of the subjects by job demand has been illustrated (Figure 1). Of the 324 subjects, 50.3% had low job demand whilst 49.7% had high job demand. The median value for job demand in the study was 14.00. The distribution of the subjects by job control has been shown (Figure 2). Of the 324 subjects, 52% had low job control whilst 48% had high job control. The median value for job control in the study was 15.00. More than a quarter (26.2%) of the subjects had high strain (Table 2).

**TABLE 2:** Distribution of subjects by perceived presence of stress at work (job strain).

Variables	*n*	%
Low strain	80	24.7
High strain	85	26.2
Active	76	23.5
Passive	83	25.6
Total	324	100.0

*n*, Given as means of number.

Amongst the pharmaceutical personnel, 45.5% were in the high strain category and of the health management and support workers, 29.9% were in the high strain group. The values amongst other health workers, laboratory personnel, nurses and Physicians were 26.9%, 25.0%, 19.8% and 11.1% respectively. This finding was not statistically significant (*p* > 0.05) (Table 3).

**TABLE 3:** Association between occupational groups and job strain.

Occupational groups	*N*	Low strain		High strain		Active		Passive		*X*^2^	*P*
			
		*n*	%	*n*	%	*n*	%	*n*	%		
Physicians	18	4	22.2	2	11.1	5	27.8	7	38.9	22.87	0.087
Nurses	81	26	31.1	16	19.8	19	23.5	20	24.7	–	–
Pharmaceutical personnel	22	4	18.2	10	45.5	6	27.3	2	9.1	–	–
Laboratory personnel	28	8	28.6	7	25.0	3	10.7	10	35.7	–	–
Other health workers	78	17	21.8	21	26.9	14	17.9	26	33.3	–	–
Health management and support workers	97	21	21.6	29	29.9	29	29.9	18	18.6	–	–

*N*, as a means of total number; *n*, as a means of number; *X*^2^, Chi-square coefficient; *p, p*-value.

The distribution of some hypertension covariates amongst job strain categories has been shown (Table 4). More than a third (38.6%) of the workers had been employed for less than 5 years, whilst 3.0% had worked for 31–35 years. The highest percentage of high strain subjects (30.9%) was seen amongst the 50–59 years age range, but there was no statistically significant association between age range and high strain in the studied population (*p* > 0.05). Male subjects had higher job strain when compared with female subjects and this was found to be statistically significant (*p* < 0.05). The alcohol consumption rate was low as the majority of the subjects did not drink alcohol. The majority of the subjects were also non-smokers. A history of alcohol consumption was statistically associated with high job strain (*p* < 0.05). There was no significant association between cigarette smoking and high job strain (*p* > 0.05). Amongst the obese subjects, 37.5% were in the high strain group, whilst amongst those with a normal body mass index, 26.2% were in the high strain group. Obesity, however, was not statistically associated with high job strain.

**TABLE 4:** Distribution of some hypertension covariates amongst job strain categories.

Variables	*N*	Low strain		High strain		Active		Passive		X^2^	*P*
			
		*n*	%	*n*	%	*n*	%	*n*	%		
**Age**											
20–29	49	18	36.7	9	18.4	8	16.3	14	28.6	13.135	0.359
30–39	90	22	24.4	23	25.6	23	25.6	22	24.4		
40–49	116	20	17.2	34	29.3	31	26.7	31	26.7		
50–59	45	14	25.5	17	30.9	12	21.8	12	21.8		
60–65	14	6	42.9	2	14.3	2	14.3	4	28.6		
**Sex**											
Male	143	30	21.0	39	27.3	45	31.5	29	20.3	11.385	0.010
Female	181	50	27.6	46	25.4	31	17.1	54	29.8		
**Physical activity of subjects**											
Yes	183	47	25.7	47	25.7	42	23.0	47	25.7	0.263	0.967
No	141	33	23.4	38	27.0	34	24.1	36	25.5		
**Use of alcohol**											
Occasional use	21	3	14.3	5	23.8	11	52.4	2	9.5	14.045	0.029
Never	228	58	25.4	57	25.0	47	20.6	66	28.9		
Ex-drinker	75	19	25.3	23	30.7	18	24.0	15	20.0		
**Smoking status**											
Daily smoker	1	6	25.0	1	100.0	9	37.5	5	20.8	9.136	0.425
Former smoker	24	1	100.0	4	16.7	67	22.5	78	26.2		
Occasional smoker	1	73	24.5	80	26.8	6	35.3	5	29.4		
Never smoked	298	4	23.5	2	11.8	48	24.6	46	23.6		
**Body mass index**											
Underweight	17	50	25.6	51	26.2	17	21.3	26	32.5	8.176	0.517
Normal	195	17	21.3	20	25.0	5	15.6	6	18.8		
Overweight	80	9	28.1	12	37.5	-	-	-	-		
Obese	32	-	-	-	-	-	-	-	-		

*X*^2^, Chi-square coefficient; *p*, *p-*value; *N*, Total number; *n*, Number of variables.

The highest percentage of hypertensives was seen amongst the high strain subjects (there were three time as many hypertensives in this group as in any other group) whilst the lowest percentage of hypertensives was seen amongst the low strain subjects, and the association between high strain and increased prevalence of hypertension was statistically significant (*p* < 0.05) (Table 5).

**TABLE 5:** Distribution of subjects by job strain and hypertension.

Job strain categories	*N*	Non-hypertensive		Hypertensive		*X*^2^	*P*
	
		*n*	%	*n*	%		
High strain	85	49	57.6	36	42.4	38.343	0.000
Passive	83	71	85.5	12	14.5		
Low strain	80	75	93.7	5	6.3		
Active	76	64	84.2	12	15.8		

*X*^2^, Chi-square coefficient; *P, p*-value.

## Discussion

The prevalence of high job strain in this study is 26.2%. One difficulty in making comparisons with other studies is that there is as yet no consensus about which absolute values should be used for defining high demand or low decision latitude. Most authors have used the median values in their own studies as the cut-off point (as was used in this study, where anyone with values above the median value was considered to suffer high job strain). Subsequently, a ‘high strain group’ might have different absolute scores in different studies.

The prevalence of high job strain is slightly higher in men (27.3%) than in women (25.4%). This was similar to what was reported from other international studies^17, 19, 20^ whilst others noted the reverse, for example, Cesana et al.^7^ found a prevalence of high job strain of 24.6% in men and 26.1% in women, in a study conducted amongst four northern Italian population samples. Whilst Omolayo and Mokuolu^21^ concluded in their research that gender is not a role determinant of job stress but that work, family, personal life and the support network of friends and co-workers, influence reactions to stress and the perception of job tension.

Although longitudinal studies are unavailable in developing countries thus far, a similar study conducted in Latin America^1^ reported the prevalence rate of high job strain across economic sectors as 26%. This data were used to estimate that high demands and low control in Latin America accounted for a range of 21% to 32% of the hypertension present in these countries.^1^

In our study, the prevalence of hypertension amongst the subjects with high job strain is 42.4%; this is quite high when compared with the prevalence amongst subjects in the other job strain categories, that is, active, passive and low strain which are 15.8%, 14.5% and 8.3% respectively. The above finding could not have been ascribed to chance as it was found to be statistically significant (*P* < 0.05). Several international studies support this finding.^17, 19, 22^ Conflicting results have been reported by Kivimäki et al.^23^ in their study in which they found no association between job strain and hypertension. They, however, advanced the reason for the negative or weak findings, which they felt, could be a consequence of the use of blood pressure measurements conducted away from the workplace, which may be less reliable and less relevant.

The highest percentage of high strain subjects was found amongst the pharmacy personnel. The above findings highlight pharmacy personnel as the occupational group with the highest percentage of subjects with high strain. The reason is not immediately clear. This finding warrants further studies because other international studies have reported associations between some types of occupation and job stress.^6^

In this study, smoking, excessive alcohol consumption, and physical inactivity were not significantly associated with high job strain, despite the commonly-held opinion that they are dangerous behaviours induced by environmental stress.^24^ Similar findings have been documented by Cesana et al. ^7^ On the contrary, Lindquist et al.^24^ suggested that work stress *per se* had no direct effect on blood pressure, but that the ways of coping with stress that individuals reported were significantly related to blood pressure, with blood pressure elevation effects apparently mediated largely by dietary, drinking habits and physical inactivity.

### Limitations of the study

In Africa, the number of studies on job stress and systemic hypertension is limited and further research is needed to better understand the mechanism and modulating factors behind the noticed relationship between job stress and systemic hypertension. It is noteworthy to mention that the use of a self-administered questionnaire to measure job stress may be subject to response bias. There is, however, no reliable objective measurement of job stress currently available.

### Recommendations

It is recommended that the management of a health workforce should ensure that the workload is in line with workers’ capabilities and abilities. Management should design jobs to provide meaning, stimulation, and opportunities for workers to use their skills. The most conscientious efforts at improving working conditions are unlikely to eliminate stress completely for all workers. For this reason, a combination of the organisational and individual approach (i.e reinforcing positive coping mechanisms such as exercise and rest) is often the most useful way to prevent or ameliorate work-related stress.

## Conclusions

The overall prevalence of job strain is 26.2% amongst the studied population. More than a quarter of the health worker population was affected, and this suggests that there is a significant level of work stress amongst the health-care workers. There is a strong association between high job strain and the presence of hypertension. This is reflected in the significantly high prevalence of hypertension (42.4%) amongst the subjects with high job strain. At the very least, it is distinctly possible that work stress is a strong contributory factor to the presence of hypertension in these subjects. Clinicians need to be aware of this association between work stress and hypertension because, when assessing patients with poor blood pressure control, the possible effect of stress on blood pressure control should be considered and appropriate measures may need to be recommended. These measures include rest, exercise and other advice on how to cope positively with stress, and in some cases redesigning a job.
